# Compton scattering imaging of a working battery using synchrotron high-energy X-rays

**DOI:** 10.1107/S1600577514024321

**Published:** 2015-01-01

**Authors:** Masayoshi Itou, Yuki Orikasa, Yuma Gogyo, Kosuke Suzuki, Hiroshi Sakurai, Yoshiharu Uchimoto, Yoshiharu Sakurai

**Affiliations:** a Japan Synchrotron Radiation Research Institute, 1-1-1 Kouto, Sayo, Hyogo 679-5198, Japan; b Graduate School of Human and Environmental Studies, Kyoto University, Sakyo-ku, Kyoto 606-8501, Japan; c Faculty of Science and Technology, Gunma University, 1-5-1 Tenjin-cho, Kiryu, Gunma 376-8515, Japan

**Keywords:** synchrotron X-ray imaging, lithium battery, *in situ* analysis, Compton scattering

## Abstract

Results of studies on Compton scattering imaging using synchrotron high-energy X-rays are reported. The technique is applied to a discharging coin cell, and the intensity of Compton scattered X-rays from the inside of the cell has been measured as a function of position and time. The position–time intensity map captures the migration of lithium ions in the positive electrode and reveals the structural change due to the volume expansion of the electrode. This experiment is a critical step in developing synchrotron-based Compton scattering imaging for electrochemical cells at a product level.

## Abstract 2

Synchrotron-based Compton scattering imaging has visualized the interior of a battery under *in situ* and *operando* conditions.

## 1. Introduction   

High-energy X-ray Compton scattering is used as the basis for an imaging technique under *in situ* and *operando* conditions. Images are obtained by position-scan measurements of Compton scattered X-rays whose intensity is proportional to the electron density in the probing volume. Since the electron density depends on constituent elements and compositions, the images can display the internal structure of an object and reveal the dynamic behaviour of materials associated with chemical reactions. The technique of Compton scattering imaging with a conventional X-ray source (Sharaf, 2001 ▶) or γ-ray source (Holt *et al.*, 1984 ▶) was demonstrated to monitor composition variations, and was applied to medical diagnosis (Guzzardi & Licitra, 1988 ▶) and analyses of archaeological and artistic objects (Harding & Harding, 2010 ▶). Because of the limited performance of those sources, however, the attained spatial resolution was sub-millimetre and the measurements suffered from low counting rates.

In this paper we demonstrate synchrotron-based Compton scattering imaging of a commercial coin cell, taking advantage of the intense highly parallel beams of synchrotron high-energy X-rays. The instrument measures the intensity of Compton-scattered X-rays from the probing volume inside the coin cell under discharge and, by scanning the collimated incident X-ray beam, an intensity map as a function of probing-volume position and discharging time is obtained. The position–time intensity map captures the migration of lithium ions in the positive electrode and reveals the change in the position of the separator due to the volume expansion of the electrode. This is the first demonstration of imaging the dynamic behaviour of materials inside a working battery using the synchrotron-based Compton scattering imaging technique.

## 2. Compton scattering imaging   

The interactions of X-rays in matter include the photoelectric effect, coherent scattering and incoherent scattering (McMaster *et al.*, 1969 ▶). Among them, Compton scattering is more important for high-energy X-rays around 100 keV compared with other interaction phenomena. Coherent scattering of X-rays with electrons depends strongly on X-ray energy and decreases as the scattering angle increases, and the photoelectric effect varies with the atomic number *Z* of materials and decreases as the X-ray energy increases. On the other hand, Compton scattering or incoherent scattering is almost constant around the X-ray energy of 100 keV and shows less dependence on scattering angle. Therefore, a simple experimental setup, such as a 90° geometry, can be used for Compton scattering imaging. For materials whose main composition is a 3*d* or lighter element, Compton scattering becomes dominant at high scattering angles.

The intensity of Compton-scattered X-rays for monochromatic X-ray beams, d*N*, is given by 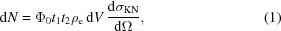
where 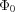 is the photon flux of incident X-rays into a target object, 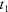 is the incident X-ray transmittance from the entrance surface to the probing volume in the object, 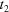 is the scattered X-ray transmittance from the probing volume to the exit surface, 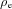 is the average electron density over the probing volume d*V*, and 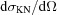 is the Klein–Nishina differential cross section (Sharaf, 2001 ▶). The probing volume d*V* is defined by a couple of slit systems for incident and scattered X-rays under a 90° scattering geometry. Since the photon flux 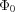 is monitored, the intensity d*N* depends on the X-ray transmittances 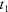 and 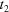, as well as the electron density 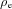. When Compton scattering is dominant and the X-ray transmittances can be treated as constants, the observed intensity d*N* represents the electron density 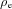 to a good approximation. As a result, an intensity map of Compton scattered X-rays can image the internal structure of an object, since the electron density depends on the material. However, for a large object or a small change in d*N* associated with a chemical reaction, the effect of the X-ray transmittances in the target object need to be considered.

One of the advantages of Compton scattering imaging over X-ray transmission imaging is the high sensitivity to the local electron density, even for a sample object surrounded by materials with high X-ray absorption coefficients. This is because Compton scattering imaging measures the X-ray intensity from a local probing volume, while X-ray transmission imaging measures the total absorptance along the X-ray path. Furthermore, Compton scattering imaging allows us to access a three-dimensional image without the need of observations from all surrounding directions. This is beneficial to imaging the internal structure and dynamic material behaviours in a large object.

## 3. Experiment and results   

**Figure 1:**
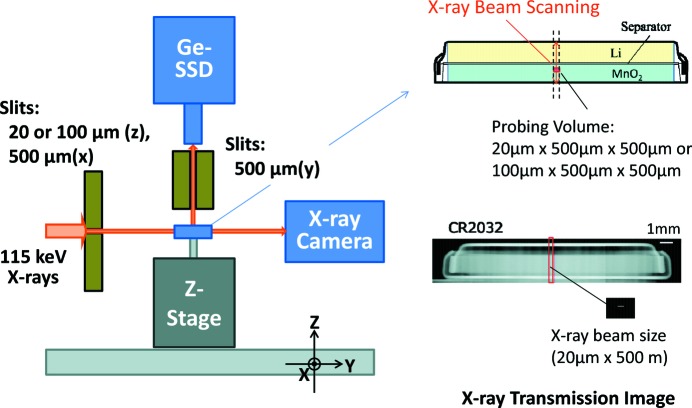
Experimental setup for Compton scattering imaging of a coin cell (CR2032). Scanning the incident X-ray beam, the intensity of Compton-scattered X-rays from a local probing volume is measured as a function of position and time. X-ray transmission images are recorded by the X-ray camera with a wide X-ray beam.

The experiment was performed at the BL08W beamline (Sakurai, 1998 ▶) at SPring-8. The X-ray source was an elliptical multipole wiggler operating in linear polarization mode (Maréchal *et al.*, 1998 ▶). Synchrotron radiation was monochromated and horizontally focused by an asymmetric Johann-type Si monochromator with (620) reflecting planes (Yamaoka *et al.*, 2000 ▶) to deliver 115 keV X-ray beams to the instrument for Compton scattering imaging. Fig. 1 ▶ shows the experimental setup for the measurements of a commercial coin cell (CR2032). The coin cell consists of a 1800 µm-thick MnO_2_ positive electrode, a 600 µm-thick Li negative electrode and a 100 µm-thick olefin-based nonwoven fabric separator. The entrance slit system upstream of the coin cell defines the size of the incident X-rays to be 20 µm or 100 µm in height and 500 µm in width. Scattered X-rays from a probing volume inside the cell are detected by a Ge solid-state detector (Ge-SSD) with a collimating slit of 500 µm at a scattering angle of 90°. Therefore, the probing volume is 20 µm or 100 µm (*z*) × 500 µm (*x*) × 500 µm (*y*). The cell is mounted on a movable *z*-stage, and the intensity of the Compton-scattered X-rays from the probing volume is measured by scanning with the X-ray beams along the vertical (*z*) direction. In addition, X-ray transmission images are taken using a two-dimensional X-ray camera (AA-40, HAMAMATSU) with non-focused wide X-ray beams and no entrance slit system.

**Figure 2:**
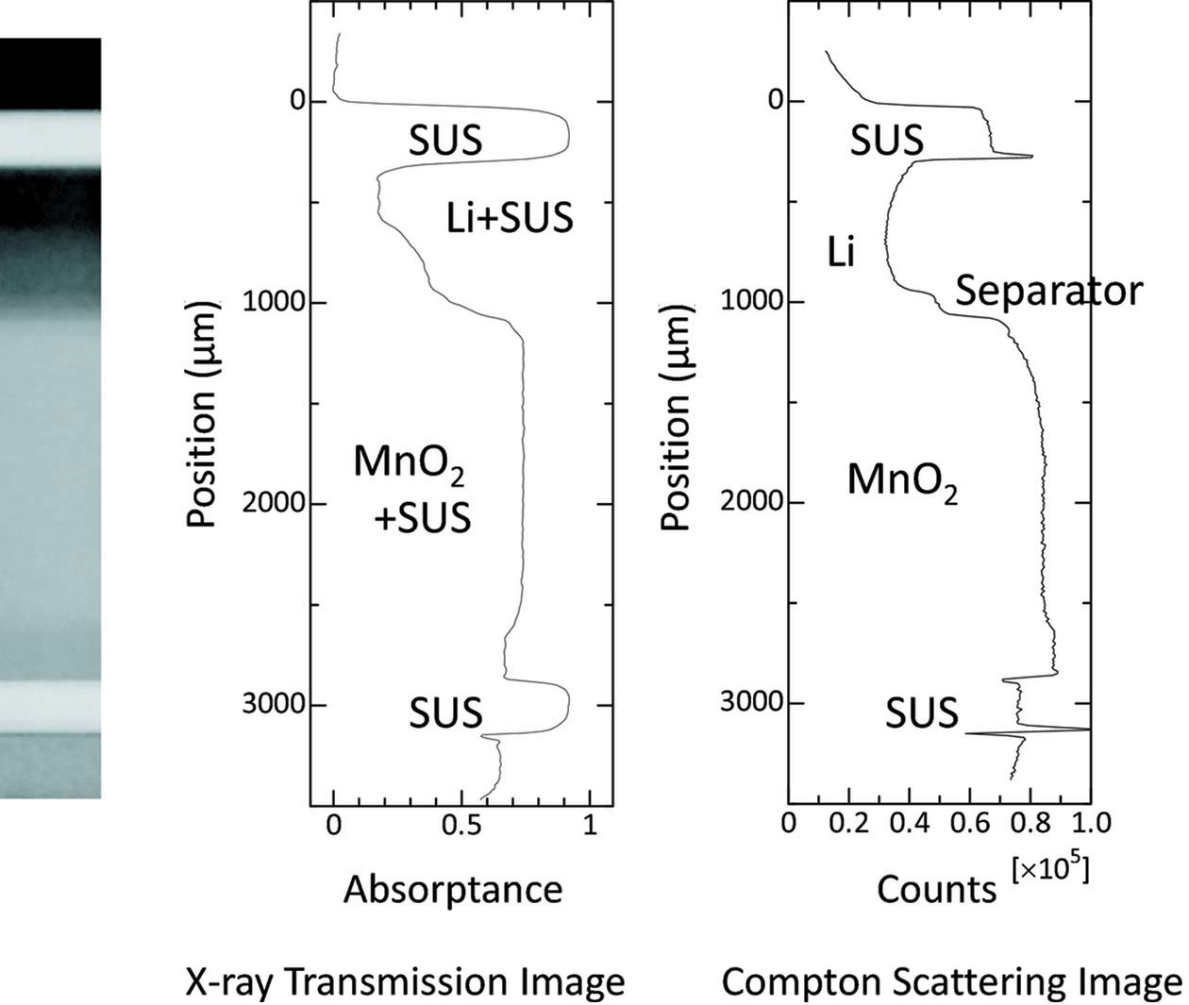
Comparison between the Compton scattering image and X-ray transmission image.

Fig. 2 ▶ shows a comparison between the Compton scattering image and the X-ray transmission image of the coin cell before discharge. The Compton scattering image was measured with a probing volume of 20 µm × 500 µm × 500 µm and a position step of 20 µm. Data were recorded for 100 s at each position. On the other hand, the X-ray transmission image was digitized from the X-ray camera data with a pixel size of 10 µm × 10 µm. Although the electrodes and the stainless steel (SUS) container are well identified in both images, the Compton scattering image is sharper than the X-ray transmission image, particularly around the separator position. The reason for this is that the Compton scattering image represents the local probing volumes, while the X-ray transmission image measures all the absorption effect along the X-ray path inside the coin cell, including the sides of the SUS container. This accessibility to a three-dimensional image from one direction is the merit of X-ray Compton scattering imaging as already mentioned above.

**Figure 3:**
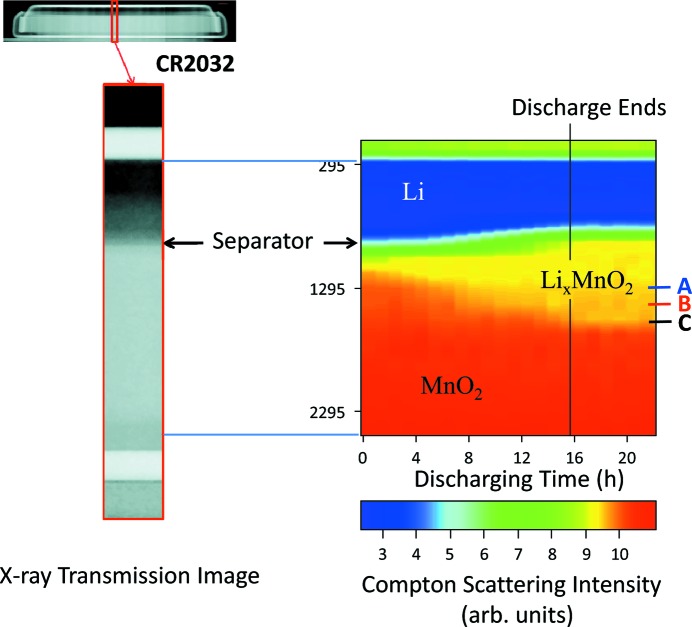
Intensity map of Compton-scattered X-rays as a function of vertical (*z*) position and discharging time of the coin cell (CR2032). The coin cell was discharged under a constant current for 15.75 h. The blue area is the negative electrode made of lithium. The red and yellow areas are MnO_2_ and Li_*x*_MnO_2_ in the positive electrode.

Fig. 3 ▶ shows the intensities of Compton-scattered X-rays from the coin cell during discharge, which were measured as a function of the *z*-position of the probing volume and discharging time. An enlarged piece of X-ray transmission image is also shown here for comparison. The vertical (*z*) position scan with 19 steps was repeated 66 times for 22 h. The data recording time was 10 s at each step and 900 s at position *A* shown in Fig. 3 ▶. The probing volume was 100 µm × 500 µm × 500 µm. The cell was discharged under a constant current (5.5 mA) for 15.75 h, and the initial voltage and end voltage were 3 V and 2 V. The current was 27.5 times larger than the standard-use current of 0.2 mA. The negative electrode made of lithium (blue area) shows less intensity compared with the positive electrode made of manganese dioxide (red area), reflecting the different electron density between the materials. The theoretical electron density of lithium metal and manganese dioxide are 3.38 and 19.8 × 10^23^ electrons/cc, respectively.

The intensity of Compton-scattered X-rays becomes weak in the positive electrode (yellow area) as the lithium ion movement proceeds, which means that the electron density decreases in the positive electrode during discharge. This is caused by the volume expansion of the electrode materials as lithium ions insert into the structural framework of manganese dioxide. This volume expansion has a larger contribution than the increase in the number of electrons by lithium ion insertion, which leads to the overall decrease of electron density. Assuming that the manganese dioxide in the cathode has a spinel structure, the discharge results in 14% increase in volume and 8% decrease in electron density [*a* = *c* = 8.0407 Å in MnO_2_ (Greedan *et al.*, 1998 ▶); *a* = 8.007 Å, *c* = 9.274 Å in LiMnO_2_ (Thackeray, 1999 ▶)].

The effect of incident X-ray transmittance 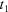 on the intensity of observed Compton-scattered X-rays is considered here. In the positive electrode, the lithium distribution changes with discharging time, leading to a change of the incident X-ray transmittance. Assuming that the lithium concentration is varied along the vertical (*z*) direction in the electrode but homogeneous on the *x*–*y* planes parallel to the incident X-ray beams, the decreased intensity of Compton-scattered X-rays indicates an increase of the incident X-ray transmittance since most of the X-ray interaction is Compton scattering. Therefore, the effect of the incident X-ray transmittance contributes to increase the observed X-ray intensity. In the present case, however, the observed result is a large decrease of the observed Compton-scattered X-rays. This fact shows that the effect of incident X-ray transmittance, and scattered X-ray transmittance as well, is small compared with that of the electron density.

**Figure 4:**
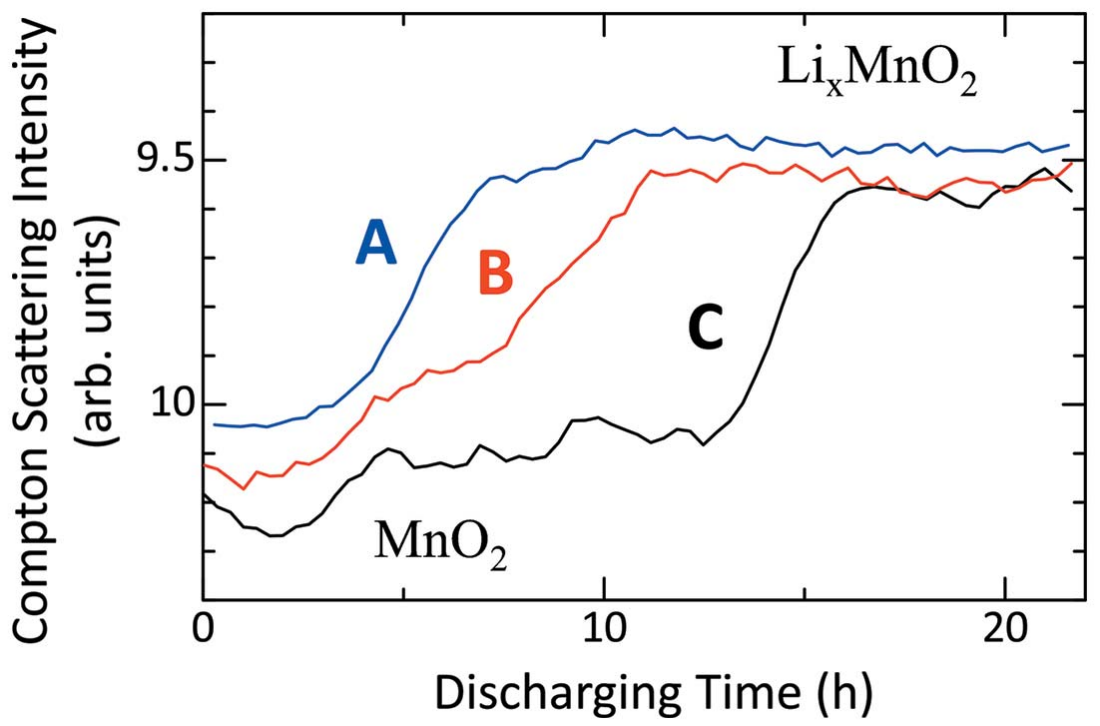
Variation of Compton-scattered X-rays with discharging time at three different positions. This displays the rate of lithium ion arrival at the positions. The three positions *A*, *B* and *C* are indicated in Fig. 3 ▶.

Fig. 4 ▶ displays the rates of lithium-ion arrival at three different positions in the positive electrode, where the positions *A*, *B* and *C* are shown in Fig. 3 ▶. All the lines start around 10 (corresponding to MnO_2_) and reach 9.5 (Li_*x*_MnO_2_) after a certain discharging time. The time difference between the intensity middle points of *B* and *C* is longer than that between *A* and *B*, indicating that the diffusion speed of lithium ions becomes slow as the position becomes further away from the separator.

**Figure 5:**
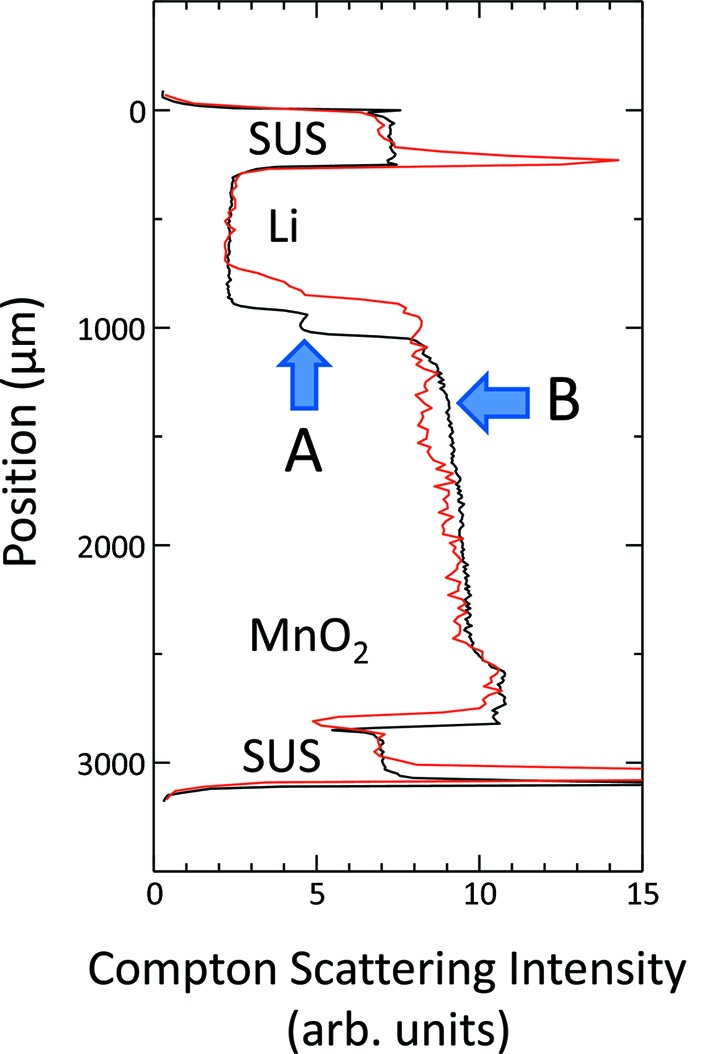
Changes in internal structure due to the expansion of the positive electrode (indicated by *A*) and lithium ion migration in the positive electrode (indicated by *B*). Black line: initial; red line: fully discharged.

Fig. 5 ▶ shows the Compton scattering images for initial and fully discharged states, with a probing volume of 20 µm × 500 µm × 500 µm and a position step of 20 µm. The data recording time at each step is 100 s for the initial state and 10 s for the fully discharged state. Two distinct differences are observed between the two states. One is the change in the separator position (indicated by *A*) due to the volume expansion of the positive electrode. The other is the change in the intensity of Compton-scattered X-rays (indicated by *B*) due to lithium ion migration and chemical reaction. In addition, the smearing of the X-ray intensity profile at the separator is observed. This means that the initially flat sheet of the separator becomes wavy at ∼500 µm after the discharge is completed. On the other hand, spike-like signals are observed at the boundaries between SUS and Li, and between SUS and the outside. These signals come from increased incident X-ray transmittance 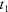 at the boundaries due to a slight inclination of the discharged cell, where Li and the outside space (air) have less X-ray absorption than SUS.

## 4. Summary   

This work has demonstrated the feasibility of synchrotron-based Compton scattering imaging for a product-level cell at work. The measurements have revealed the intensity variation of Compton-scattered X-rays, which reflects the internal structure change and the lithium ion migration during discharge. Direct observations of electrochemical processes and internal structure changes are crucial to developing advanced batteries. In particular, the interplay between them under working conditions is critical to the design of battery cells. This method is applicable to other electrochemical devices, such as various rechargeable batteries and fuel cells.

This work is a critical step for further developments. We are developing a compound refractive nickel lens for high-energy X-rays to increase the incident X-ray photon flux density for smaller probing volumes (Andrejczuk *et al.*, 2014 ▶) and a new analytical method for Compton-scattered X-rays in order to overcome the X-ray transmittance problem for large objects (Suzuki *et al.*, 2015 ▶).
